# Rough Edges, Meticulous Attention

**DOI:** 10.3201/eid3012.AC3012

**Published:** 2024-12

**Authors:** Byron Breedlove

**Keywords:** Nebamun fowling in the marshes, Egyptian tomb art, zoonotic infections, malaria, toxoplasmosis, schistosomiasis, leishmaniasis, zoonoses, art and science, public health, about the cover

**Figure:**
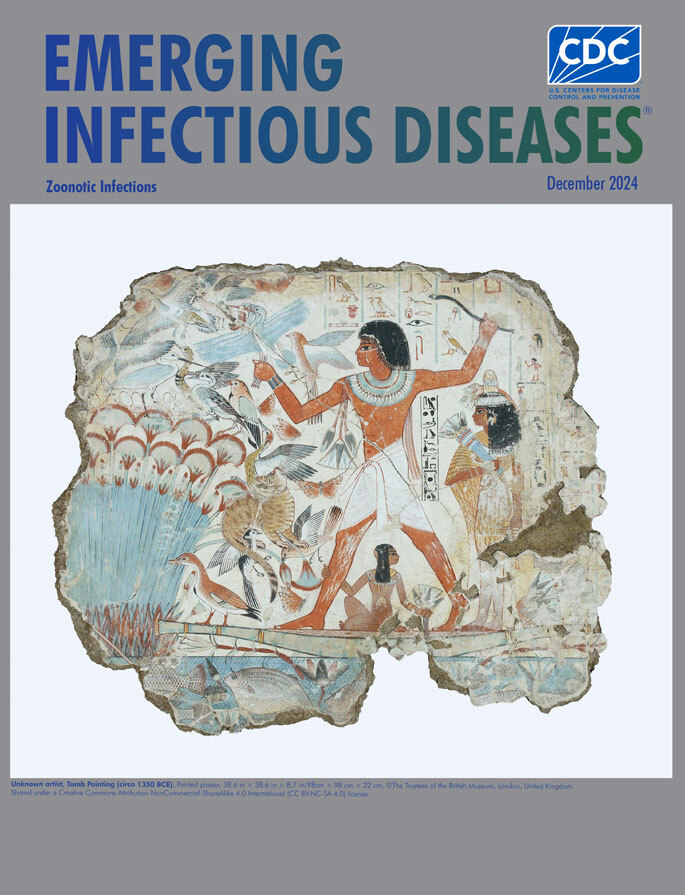
**Unknown artist, Tomb painting identified as Nebamun Fowling in the Marshes fragment (circa 1350 BCE).** Painted plaster, 38.6 in × 38.6 in × 8.7 in/98 cm × 98 cm × 22 cm. © Trustees of the British Museum, London, United Kingdom. Shared under a Creative Commons Attribution-NonCommercial-ShareAlike 4.0 International (CC BY-NC-SA 4.0) license.

This month’s cover features a well-known example of ancient Egyptian tomb art. The tomb walls were first plastered with a thick mixture of mud and straw, followed by a thinner, smooth layer suitable for painting. According to the British Museum, a team of artists created the images, initially sketching outlines for each scene, then adding details, using for pigments a range of natural materials, including soot and various ground stones and minerals. Despite being more than 3,000 years old, the paintings are well-preserved, in measure because of the dry air in the sealed tomb.

This painting, which is known as Nebamun Fowling in the Marshes, is among the artifacts that antiquities hunters removed from the tomb chapel of Nebamun in 1820.^1^ Richard Parkinson, professor of Egyptology and curator with the British Museum, explains that archaeological methods and practices were then quite primitive and the workers who removed the art “probably cut into the plaster surface of the walls with knives and saws, outlining rectangular pieces which they then pried off the walls with implements such as crowbars. Sometimes they removed the full depth of the mud plaster, but sometimes they carried away only a very thin layer, and the edges inevitably suffered cracking and disintegration.”

The British Museum, which subsequently acquired many of those artifacts, notes that “The wall paintings from Nebamun’s tomb chapel show an idealised vision of daily ancient Egyptian life,” and adds that “Nebamun’s tomb chapel was a place for people to come and commemorate Nebamun and his wife after his death with prayers and offerings. Nebamun himself was buried somewhere beneath the floor of the innermost room of the tomb chapel in a hidden burial chamber.”

Nebamun, the subject of these paintings, has been identified as a wealthy government official who served as an accountant and scribe overseeing the collection and accounting of grain supplies. This marsh hunting scene depicts him standing on a small boat hunting birds, gripping a throw stick on one hand and decoy herons in the other. His young daughter, sitting beneath him on the skiff, is shown clutching his leg. Nebamun’s wife, Hatshepsut, dressed in finery, stands behind him. Parkinson wrote that “With his black wig and beaded collar, holding his snake-headed throwing stick, he strikes an athletic, dynamic, almost heroic pose, as master of the whole proceedings.”

The marsh teems with wildlife. Fish swim below the part of the waterline that remains intact. An array of startled birds emerging from a thicket of papyrus fills the left side of the panel. Several lotus flowers, considered symbols of rebirth and everlasting life, appear. A tabby cat capturing one bird in its claws and another with its teeth shows that Nebamun is not the only hunter here. The British Museum also notes that the gold leaf gilding on the cat’s eye is the only example of such gilding to be found on wall paintings in Theban tomb chapels. Details, such as finely rendered scales, speckles, feathers, and fins attest to meticulous attention by the artists. The British Museum notes that the painting also includes “remains of eight vertical registers of hieroglyphs” visible on the right half of the panel.

Nebamun’s cause of death is not known or suggested by any of the extant rough-edged panels from his tomb. After Giovanni d'Athanasi discovered the tomb in 1820, he kept the tomb’s exact location, somewhere on the west bank of the Nile, secret because he wished to protect it from rival antiquities hunters and because of a dispute with Henry Salt, the British Consul General in Egypt who had hired him. What else might remain in the tomb, including clues about Nebamun’s cause of death, continues to be a mystery. A recently published meta-analysis by Piers D. Mitchell in *Advances in Parasitology* examines evidence of parasitic and zoonotic infections found in Egyptian mummies, and considering his research, some of the imagery from this panel could tempt conjecture. For instance, could the prominently featured tabby cat have transmitted toxoplasmosis to Nebamun? Was he possibly infected with malaria transmitted by mosquitos that flourished near the Nile River or schistosomiasis acquired from contaminated fresh water in the marsh? Might he have acquired leishmaniasis after being bitten by infected female sand flies?

Although such questions cannot be answered, the scope and impact of zoonotic infections are well known. According to the Centers for Disease Control and Prevention, “Scientists estimate that more than 6 out of every 10 known infectious diseases in people can be spread from animals, and 3 out of every 4 new or emerging infectious diseases in people come from animals.” A number of articles in this issue of *Emerging Infectious Diseases* document outbreaks and case reports of zoonotic diseases emerging around the world. Applying the same sort of meticulous attention to detail that those ancient artists lavished on Nebamun’s tomb paintings is a crucial component of the public health efforts to mitigate such diseases.

## Footnotes

1 In *The Painted Tomb-Chapel of Nebamun*, Richard Parkinson, discussed how the British Museum acquired this panel and others from Henry Salt, who was the British consul-general in Egypt. Salt employed agents, including Giovanni d'Athanasi, who discovered Nebanum’s tomb, to excavate and remove objects. Parkinson briefly noted how antiquity hunters competed among each other and kept locations of discoveries secret, a practice that has obscured the provenance of these panels and of other items.
